# Vitamin K and Calcium Chelation in Vascular Health

**DOI:** 10.3390/biomedicines11123154

**Published:** 2023-11-27

**Authors:** Jan O. Aaseth, Urban Alehagen, Trine Baur Opstad, Jan Alexander

**Affiliations:** 1 Research Department, Innlandet Hospital Trust, P.O. Box 104, N-2381 Brumunddal, Norway; 2 Faculty of Health and Social Sciences, Inland Norway University of Applied Sciences, P.O. Box 400, N-2418 Elverum, Norway; 3 Division of Cardiovascular Medicine, Department of Medical and Health Sciences, Linköping University, SE-581 85 Linköping, Sweden; urban.alehagen@liu.se; 4 Oslo Centre for Clinical Heart Research Laboratory, Department of Cardiology, Oslo University Hospital Ullevål, P.O. Box 4950, Nydalen, N-0424 Oslo, Norway; t.b.opstad@medisin.uio.no; 5 Faculty of Medicine, University of Oslo, N-0370 Oslo, Norway; 6 Norwegian Institute of Public Health, P.O. Box 222, N-0213 Oslo, Norway; jan.alexander@fhi.no

**Keywords:** vitamin K, chelation, matrix Gla protein, osteocalcin, EDTA, vascular calcification, bone loss

## Abstract

The observation that the extent of artery calcification correlates with the degree of atherosclerosis was the background for the alternative treatment of cardiovascular disease with chelator ethylenediamine tetraacetate (EDTA). Recent studies have indicated that such chelation treatment has only marginal impact on the course of vascular disease. In contrast, endogenous calcium chelation with removal of calcium from the cardiovascular system paralleled by improved bone mineralization exerted, i.e., by matrix Gla protein (MGP) and osteocalcin, appears to significantly delay the development of cardiovascular diseases. After post-translational vitamin-K-dependent carboxylation of glutamic acid residues, MGP and other vitamin-K-dependent proteins (VKDPs) can chelate calcium through vicinal carboxyl groups. Dietary vitamin K is mainly provided in the form of phylloquinone from green leafy vegetables and as menaquinones from fermented foods. Here, we provide a review of clinical studies, addressing the role of vitamin K in cardiovascular diseases, and an overview of vitamin K kinetics and biological actions, including vitamin-K-dependent carboxylation and calcium chelation, as compared with the action of the exogenous (therapeutic) chelator EDTA. Consumption of vitamin-K-rich foods and/or use of vitamin K supplements appear to be a better preventive strategy than EDTA chelation for maintaining vascular health.

## 1. Introduction

Early studies identified calcification in the media and intima of coronary arteries to be associated with arterial stiffness and increased risk for adverse cardiovascular events [1,2]. The extent of artery calcification was further reported to be correlated with the degree of atherosclerosis and with the risk of cardiovascular events [3,4]. The possible pathogenic role of arterial calcium deposits in cardiovascular disease (CVD) led to the early use of infusions of the chelating agent ethylenediamine tetraacetate (EDTA) to dissolve these precipitates [5]. EDTA is a chelating drug that binds calcium and several other metal cations, including magnesium and zinc, thereby facilitating their urinary excretion [6]. However, EDTA treatment may be associated with severe side effects, such as hypocalcemia and hypomagnesemia [7]. In this context, the function of non-toxic endogenous calcium chelating agents is of great interest, i.e., the proteins osteocalcin and matrix Gla protein (MGP), which are activated through vitamin-K-dependent enzymatic carboxylation [8]. Thereby, their glutamate residues are transformed into dicarboxylic acids with high affinity for calcium ions [9]. Activated MGP with its five chelating groups operates as an inhibitor of arterial calcification and may thereby delay an atherosclerotic process [10]. The cooperation with osteocalcin with three dicarboxylic groups is essential for the transfer of calcium into the bones [10,11]. A key function of vitamin K is to catalyze the carboxylation of specific glutamic acid residues in some vitamin-K-dependent proteins (VKDPs), including osteocalcin and matrix Gla protein (MGP), as well as several other proteins [12], i.e., several factors involved in blood clotting. Low plasma concentrations of clotting factors II and VII have been associated with increased risk of cardiovascular mortality in elderly with signs of heart failure [13]. It is worth noting that vitamin K may also work via a nuclear receptor as a specific form of vitamin K, i.e., menaquinone 4 (MK4), may activate the steroid and xenobiotic receptor (SRX), the human analogue of the pregnane and xenobiotic receptor (PXR) in rodents [14].

Activated MGP is a potent inhibitor of vascular calcification, while osteocalcin is essential for bone mineralization [15,16].

The importance of vitamin K in cardiovascular and bone health has been extensively studied in recent years, and there is growing evidence to support its role in the prevention of vascular calcification [17]. Vascular and valvular calcification with deposition of calcium in blood vessel walls and heart valves leads to increased stiffness and reduced flexibility of the vessels and valves, thus representing a significant risk factor for cardiovascular morbidity and mortality [18]. The clinical evidence supporting the role of vitamin K in the delay of calcification is increasing [19]. This has led to a growing interest in the potential use of vitamin K supplements in the prevention of CVD. Nevertheless, it must be admitted that the mechanisms of action and the clinical role of the different forms of vitamin K are still insufficiently known, which has precipitated the present article.

This review aims to provide an overview of the role of vitamin K in cardiovascular health, with a particular focus on the kinetics of different forms of vitamin K and the mechanisms of vitamin-K-dependent carboxylation and calcium chelation. The advantages of the endogenous chelators over exogenous chelators such as EDTA are outlined. The review discusses the clinical evidence supporting the role of vitamin K in preventing CVD, as well as potential future directions for research in this field.

## 2. Sources and Kinetics of Vitamin K

Different chemical forms of vitamin K have been used in previous studies and reviews on impacts of the vitamin on vascular calcification and other health effects. Here, we briefly describe the different forms of vitamin K; dietary sources; and their absorption, metabolism, and possible differences related to their role in vascular calcification.

**Figure 1:**
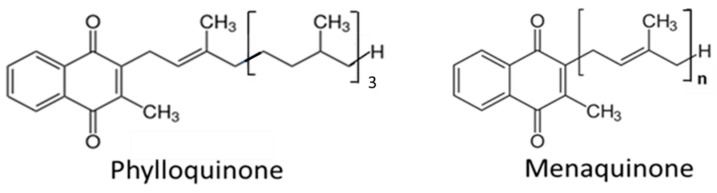
The structure of vitamin K1 (phylloquinone) and vitamin K2 (menaquinone) with the number n of isoprenyl groups at its C3 position.

Natural vitamin K exists as two vitamers: vitamin K1 (known as phylloquinone (PK)) found in leafy green vegetables [20] and vitamin K2 (a group of menaquinones, often denoted MKs) (Figure 1) found in fermented foods and foods of animal origin [21]. Vitamin K also exists in the gut microbiota to an unknown extent, formed by intestinal bacteria [22,23]. Vitamin K2 occurs both as short-chain menaquinone-4 (MK4) and as long-chain menaquinone-7, -8, and -9 (MK7, -8 and -9), where the numbers denote the number of isoprenyl groups at the C3 position (see Figure 1). The naphthoquinone ring without the side chain is denoted menadione or vitamin K3 [23].

Following incorporation into mixed micelles of bile salts, triglycerides, and phospholipids, vitamin K is absorbed in the proximal part of the small intestine. The absorption is mediated by several transporters including the Nieman-Pick C1-Like 1 (NPC1L1), which also transports cholesterol and vitamin E [23,24]. Of note, ezetimibe, which is used for blocking NPC1L1-mediated cholesterol uptake from the gut, also inhibits vitamin K uptake [23]. While this cholesterol transporter appears to be clinically important, less is known about the role of other cholesterol and vitamin E transporters, except for scavenger class B type I (SR-BI) and CD36, which both appear to be involved in vitamin K absorption. Following absorption, vitamin K is mainly found in triglyceride-rich lipoproteins, which are rapidly cleared by the liver [25]. MKs may also occur in low- and high-density lipoproteins. The plasma levels of PK, which do not directly reflect PK intake, are mainly influenced by the plasma triglyceride level [26]. The knowledge on transporters has primarily been obtained via the uptake of PK, and less is known about the uptake mechanisms for MKs (vitamin K2) [23]. It is, however, likely that due to structural similarity and lipophilicity, MKs are using the same transporters as vitamin PK. The synthesis of MKs by intestinal bacteria mainly occurs in the colon, and due to the absence of bile salts, which are needed for micelle formation and thus absorption, their contribution is uncertain [27].

**Figure 2:**
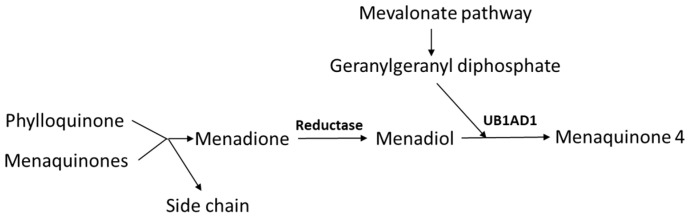
Pathway of endogenous menaquinone 4 (MK4) synthesis. The side chain is split off during intestinal absorption, while reduction in menadione and prenylation is taking place in the tissues (UB1AD1 denotes UbiA Prenyltransferase Domain-Containing Protein 1).

In addition to the dietary intake of MKs, PK can be converted to MK4 [28]. Side-chain removal with the release of menadione seems only to take place in the intestines during absorption, as the release of menadione was absent upon parenteral administration of PK [29,30]. In extrahepatic tissues, the released menadione is reduced to menadiol, and subsequently prenylated by the UB1AD1-enzyme (UbiA Prenyltransferase Domain-Containing Protein 1) to MK4, using geranylgeranyl diphosphate as co-substrate (Figure 2). Recent experiments in rodents have shown that also the side chains of dietary MK7 and MK9 are in part removed resulting in extrahepatic MK4 synthesis [31]. Menadione appears to be a systemic precursor for MK4 formation in the tissues [30], and MK4 is formed both upon oral and parenteral administration of menadione.

The formation of the polyisoprenyl side chain of MK4 is dependent on a functional mevalonate pathway, which can be blocked by the 3-hydroxy-3-methylglutaryl CoA (HMGCoA) reductase inhibitory statins that are used to lower cholesterol synthesis [32]. Also, bisphosphonates, used in the treatment of osteoporosis, may interfere with this pathway by inhibiting farnesyl-diphosphate formation [33,34].

**Figure 3:**
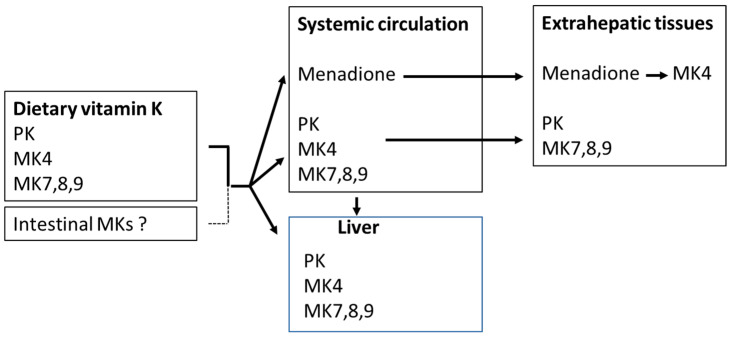
Uptake and distribution of phylloquinone (PK) and menaquinones (MKs). Menadione is formed by side-chain cleavage of dietary phylloquinone and menaquinones.

The organ distribution of PK and MKs differs (Figure 3). MK4 is present in the kidney, brain, bone, skin, and exocrine and endocrine glands [16], and to a lesser extent in the liver. MK7 is mainly found in the liver, spleen, adrenal glands, and testes, whereas PK is predominantly found in the liver and small intestine [35]. Upon oral administration, it appears that long-chain menaquinones (MKs) reach higher plasma concentrations and have considerably longer elimination half-lives than equimolar amounts of phylloquinone (PK) or MK4 [8,36,37]. However, in plasma, PK is usually the dominating species, while MK4 is either undetectable or occurs in low concentrations, and the MK7 concentration is dependent on the dietary intake of fermented food or supplementation [38,39].

## 3. The Vitamin-K-Dependent Carboxylation, the Vitamin K Cycle, and Pharmaceuticals Affecting Vitamin K

**Figure 4:**
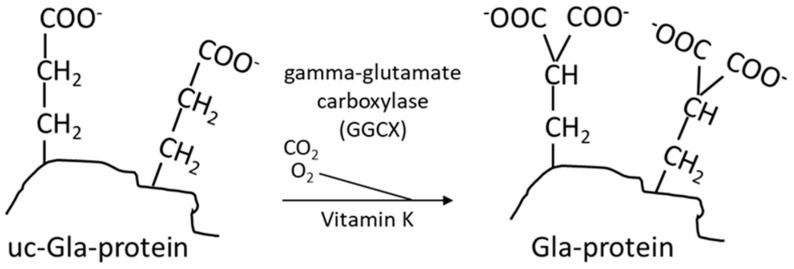
Vitamin-K-dependent carboxylation of a glutamic acid containing protein (Glu protein) to a protein containing two carboxyl groups (Gla protein). These two carboxyl groups confer chelation property to the protein [6].

**Figure 5:**
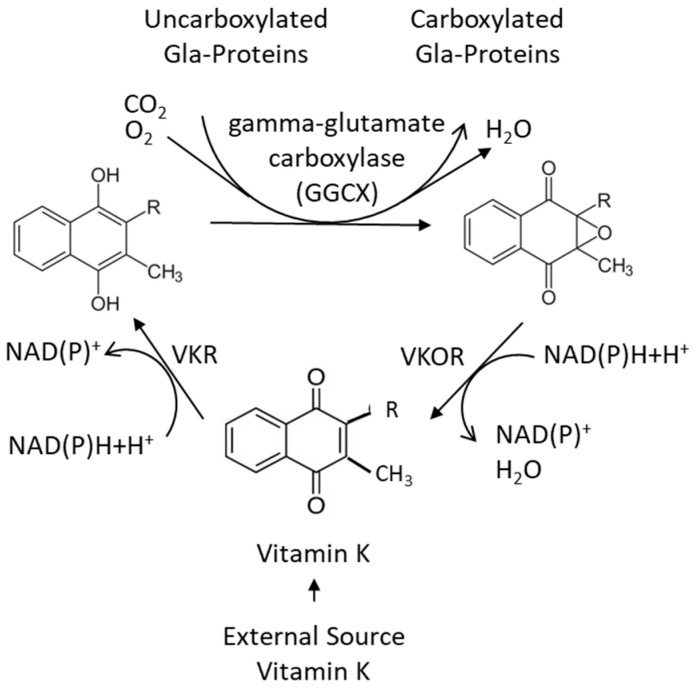
The vitamin K cycle, in which the oxidized vitamin K is reduced and reactivated [42].

Vitamin K is, as mentioned above, an essential nutrient required as a cofactor for γ-glutamylcarboxylase (GGCX), which is responsible for the carboxylation of vitamin-K-dependent proteins (VKDPs) [40] (Figure 4). The naphthoquinone ring of both vitamin K1 and K2 is reduced to an active form called vitamin K hydroquinone (Figure 5), which is the cofactor required for the carboxylation of VKDPs.

Vitamin-K-dependent carboxylation is a vital process that plays a critical role in maintaining vascular and bone health and in physiological blood clotting [41]. It involves a post-translational modification with the addition of carboxyl groups (COOH) to specific glutamic acid residues in VKDPs. The carboxylation reaction consists of conversion of specific amino acid residues from glutamic acid (Glu) to γ-glutamylcarboxylated amino acids (Gla) (Figure 4), which is essential for the calcium binding capacity of these proteins. Although the organ distribution of PK and MKs differs, there is little evidence at present indicating that PK and MKs differ in their ability to support the carboxylation of hepatic and extrahepatic VKDPs [27].

VKDPs such as MGP and osteocalcin play a crucial role in regulating the calcium traffic in the body. In the carboxylation reaction, carbon dioxide (CO_2_) and oxygen (O_2_) represent essential substrates by being sources for the addition of the extra carboxyl group to the protein, while vitamin K is converted from its reduced form to the epoxidized vitamin [42]. This activating step is crucial for the proteins to tightly bind calcium ions into chelates, which is necessary for their ability to carry calcium from the vascular space to the bones. Thereby, MGP in its activated form is a potent inhibitor of arterial calcification, as it is highly expressed in healthy arteries. Presumably, the phosphorylation of two serin units in MGP contributes to the affinity and selectivity of the protein to calcium ions [8]. Vitamin K deficiency results in an undercarboxylated and inefficient MGP. The same deficiency affects the activation of other VKDPs including osteocalcin. As osteocalcin is a bone-specific protein that is crucial in bone mineralization, vitamin K deficiency has been associated with decreased bone mineral density and an increased risk of fractures [43]. It has been suggested that vitamin K deficiency after bariatric surgery can contribute to the development of postoperative osteoporosis despite adequate vitamin D supplementation [44].

The conversion of vitamin K to vitamin K hydroquinone, as well as the reduction in the vitamin K epoxide, depends on the availability of reducing equivalents, such as NAD(P)H. While vitamin K epoxide reductase (VKOR) has been characterized, vitamin K reductase (VKR) has not [42,45,46]. It has been shown that both VKOR and NAD(P)H quinone oxidoreductase1 (NQO1) (previously DT-diaphorase) may possess some VKR activity, but none of these appear to account for all the VKR activity [40] (Figure 5).

The importance of vitamin K in carboxylation is underlined by the fact that its deficiency, by leading to an impaired carboxylation and reduced activity of VKDPs, can lead to health problems, such as bleeding disorders [47] and impaired cardiovascular health [48]. Maintaining adequate levels of vitamin K through a balanced diet or supplementation is essential for optimal carboxylation and overall health. However, it should be noted that certain medications can interfere with vitamin K function and carboxylation and affect its activity, such as the commonly used anticoagulant drug warfarin that inhibits VKOR in the vitamin K cycle and promotes vascular and valvular calcification [49], and ezetimibe that inhibits the intestinal uptake of vitamin K [50]. Although the role of statins that may inhibit MK4 synthesis appears more unclear [32,51], one might envisage that high-intensity statin treatment, particularly when combined with ezetimibe, despite its proven beneficial effects on atheroma progression, might cause detrimental effects on vascular calcification [52]. Further studies are needed to clarify these issues [53,54].

## 4. Vitamin K, Human Steroid and Xenobiotic Receptor (SXR), and Inflammation

Another mechanism by which vitamin K can affect vascular calcification is interaction with the SXR nuclear receptor (or its rodent homologue, the pregnane xenobiotic receptor (PXR)) [14]. SXR/PXR heterodimerizes with retinoid X receptor (RXR) after ligand binding, and MK4 is the only vitamin K homologue that binds to and activate SXR/PXR [55]. In osteoblasts, MK4 has been shown to activate the classical SXR target CYP3A4, in addition to several genes involved in bone formation, i.e., bone-specific alkaline phosphatase (ALP), osteopontin (OPN), matrix gla protein (MGP), osteoprotegrin (OPG), and other matrix-related genes [56,57]. It appears that SXR/PXR plays an important role in bone maintenance as PXR knockout mice show enhanced bone resorption and develop severe osteopenia despite adequate dietary vitamin K [58].

Regarding vascular calcification, the role of MK4 and SXR is less clear. Contrary to expectations, MK4 via SXR activation, in a high phosphate medium, appeared to accelerate warfarin calcification in human aortic valve interstitial cells from patients with calcified aortic valve stenosis [59], while in cells taken from non-calcified aortic valves, the SXR agonist warfarin did not cause calcification [60]. At present, it is not possible to conclude on the precise role of MK4 activation of SXR in vascular calcification.

Atherosclerosis is a chronic inflammatory process in the vascular wall where proinflammatory cytokines secreted from activated macrophages may promote osteogenic transition of vascular smooth muscle cells via NF-κB signaling, and as vitamin K has been shown in experimental studies to inhibit NF-κB signaling, this might indicate yet another mechanism of vascular protection [61].

## 5. Calcium Chelation Therapy—With Endogenous or Exogenous Chelators

Calcium chelation is a process by which calcium ions are bound to a chelating agent, for instance, to vitamin-K-activated proteins. Chelating agents can form bonds with metal ions, such as calcium, lead, and cadmium, and thereby lower their levels in the circulation [62]. Endogenous calcium chelation by MGP and osteocalcin appears to represent essential physiological processes for maintaining cardiovascular and bone health. Excess calcium deposited in blood vessels can lead to vascular calcification and loss of elasticity as well as calcification of vascular plaques, and thereby aggravate CVD especially in older individuals with an increased vascular calcification risk [48,63,64]. Carboxylation of MGP allows it to bind calcium upon its transfer to the blood vessel walls, preventing the ions from deposition and causing calcification [63]. Furthermore, MGP may also work by inhibiting bone morphogenic protein-2 (BMP-2) that promotes the osteogenic transition of vascular smooth muscular cells [65,66]. Studies have shown that an increased fraction of undercarboxylated MGP is associated with an increased risk of vascular calcification and CVD [63]. Activated MGP facilitates the export of the calcium ions from the vascular system to the bone tissue, where calcium is needed for mineralization. Complete activation of MGP also requires phosphorylation of its two serine residues that may further contribute to its calcium affinity and its ability to carry calcium ions to the bone tissue [8]. Increasing evidence regarding the importance of VKDPs in maintaining vascular health has been obtained in recent years [12,63]. It has also been reported that VKDP GAS6 (growth arrest-specific protein-6) inhibits the calcification of blood vessels and the apoptosis of vascular smooth muscle cells [12]. Taken together, current research suggests that vitamin K plays a crucial role in maintaining vascular as well as bone health, although the mechanisms of vitamin K in calcium chelation still deserve to be studied [67]. In future studies on the proposed decalcification by vitamin K supplementation, assessments of calcium in coronary arteries and aortic valve should be included among the primary endpoints.

**Figure 6:**
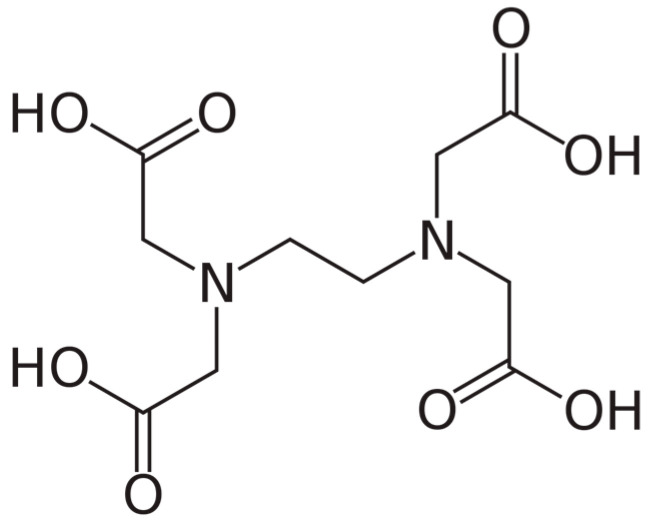
Chemical structure of ethylenediaminetetraacetic acid (EDTA), a chelating agent with four carboxylic groups.

As regard therapeutic calcium chelation, the *alternative* therapy of atherosclerosis with the exogenous chelator EDTA (Figure 6) is briefly commented in the following paragraphs.

EDTA was first launched in medicine for the treatment of lead poisoning [68], and it has later been used as an antidote in various other heavy metal poisonings [6]. In such cases, it is usually administered as a calcium complex (Ca-EDTA) to avoid hypocalcemia as a side effect. However, the clinical use of EDTA involves the risk of disruption of the physiological functioning of several metal ions in biological systems. For this reason, the use of EDTA for treatment of severe CVD has been discouraged [7].

## 6. The Use of EDTA Chelation Therapy in the Context of Vascular Health

Although EDTA in clinical use was reserved for heavy metal poisonings, the same drug has been promoted as a treatment for CVD [69,70,71]. This chelation therapy for atherosclerosis is still practiced around the world as an alternative treatment. The therapy involves multiple intravenous infusions of solutions of EDTA, believed to remove calcium from deposits in blood vessel plaques [69]. EDTA treatment has been offered as a way of breaking down the blockages in arteriosclerotic blood vessels. However, there were early concerns about the potential negative impact of EDTA, as it removes magnesium and other essential metals from the body [72]. A Cochrane review from 2020 concluded that there is limited high-quality research and evidence-based medicine on the topic, specifically regarding clinical outcomes [73], although all previous studies were considered [74,75,76,77]. In addition, the more recently presented TACT study (Trial to Assess Chelation Therapy) [70] only reported a tendency to benefit as regard their predefined composite endpoint. The primary endpoints in the TACT study were a composite of death from any cause, reinfarction, stroke, coronary revascularization, or hospitalization for angina. A post hoc analysis of the TACT study including only the participants with diabetes and peripheral artery disease reported that the EDTA therapy reduced the occurrence of primary endpoints in this subgroup as compared with diabetic patients given placebo [78]. However, this effect might be due to the addition of glucose (1.2%) in the placebo solution, which was not added to the EDTA solution [70]. Their observations are being investigated further in ongoing trials (TACT2) [71], suggesting chelation of lead and cadmium as a possible cause of the hypothesized therapeutic action [71,79]. Protocol discrepancies in the initial TACT study have been commented [80], and the researchers had no information on the vitamin K status of their participants. A *JAMA* editorial concluded that the findings of TACT should not be used as a justification for increased use of this controversial therapy [81].

However, a mechanism of the suggested positive effects of EDTA [78] might reside in some delay of ongoing plaque formation or a stabilization of existing plaques. Anyhow, if chelation therapy really stabilizes calcium plaques in vessel walls, it is tempting to suggest that an activated MGP obtained by adequate vitamin K intake may represent a more tolerable alternative than multiple EDTA infusions. Studies have suggested that long-term EDTA chelation with increased calcium loss from the body can cause hypocalcemia and increased release of parathyroid hormone (PTH) [82], which is a well-known cause of bone loss and osteoporosis [83]. Furthermore, EDTA infusions may be associated with hypomagnesemia [7], which involves the risk of cardiac arrhythmias [84]. An apparent alternative to EDTA chelation is, therefore, the optimization of natural chelators, such as VKDPs including MGP and osteocalcin. Future studies should investigate the potential synergistic effects of combining vitamin K mediated chelation therapy with other pharmacological interventions.

## 7. Clinical Evaluations of Vitamin K Supplementation

MGP and GAS6 are expressed in various tissues, including in arterial walls. In activated forms, these proteins are considered to act as inhibitors of arterial calcification and presumably also as plaque stabilizers [85]. The binding of these endogenous chelators to ions of calcium prevents their deposition in the arterial wall, thus preserving arterial elasticity and preventing the development of vascular calcification [86]. The formed calcium–MGP complex is removed from circulation and degraded by the liver, releasing the calcium moiety and promoting its deposition in the bone [12]. Animal studies have demonstrated the critical role of MGP in preventing vascular calcification, as mice deficient in MGP showed accelerated arterial calcification and increased mortality compared with wild-type mice [61]. In a series of studies in humans, a reduction in plasma dephospho-uncarboxylated MGP, a biomarker of vitamin K status, has been documented following vitamin K supplementation [87,88]. Human observational studies have also revealed that low vitamin K status is associated with an increased CVD risk, independent of other risk factors, while in supplementation studies, vitamin K decreases vascular calcification. Studies in humans are discussed in the following paragraphs.

Already in 2009, Gast et al. reported that in a prospective cohort (Prospect-EPIC cohort) including 16,057 women, a high menaquinone intake was associated with reduced incidence of coronary heart disease during a mean follow-up time of 8.1 years [89]. The intake of vitamin K1 and K2 (mean values: 212 and 29.1 μg/day, respectively) was estimated from a food frequency questionnaire (FFQ). The researchers found an inverse relationship between vitamin K2 and CVD, with a reduction in the hazard ratio by 9% for each additional 10 µg vitamin K2 dose. No association was found for vitamin K1 [89].

In 2016, Vissers et al. investigated the association of vitamin K1 and K2 intake, as estimated by an FFQ, with peripheral artery disease (PAD) incidence in a prospective cohort study of 36,629 participants. During 12 years of follow-up, 489 incident cases of PAD were documented using national registries. When comparing the highest quartile of the intake of menaquinones with the lowest, a 30% reduced risk of PAD hazard ratio (HR) 0.7 was observed. An even stronger association was observed in participants with hypertension who got a reduction in the risk by 49%, HR = 0.59, and in patients with diabetes with a reduced risk of HR = 0.56. Phylloquinone intake was not associated with significantly reduced PAD [90].

In 2019, a review article by Ruiz-Leon et al. [91] reported that, in several studies, high intake of vitamin K2 was associated with a lower risk of vascular calcification and CVD. In the Multi-Ethnic Study of Atherosclerosis, a case-control sub-study showed that in a subgroup receiving anti-hypertensive medication, low serum vitamin K1 concentrations were associated with greater progression of coronary artery calcification measured with computer tomography [92].

In the Hordaland Health Study, a prospective cohort study, 2987 healthy Norwegian subjects without CVD (aged 46 to 49 years, 43% men) were followed up for 11 years. A reduced risk of incident CVD was associated with increased vitamin K2 intake, but not with K1, as estimated from a food frequency questionnaire. The relationship was weakened upon adjustment for dietary confounders [93].

In the Danish Diet Cancer and Health study, comprising 53,372 citizens [94], dietary vitamin K, both K1 and K2, was associated with a reduced risk of hospitalization for atherosclerotic CVD during 21 years of follow-up. Atherosclerotic CVD included ischemic stroke, ischemic heart disease, and PAD. For all outcomes, there were significant associations between the intake of both vitamin K1 and vitamin K2 and a reduced risk. Dietary vitamin K intake was estimated from validated food frequency questionnaires. In another study performed in the same cohort comprising 56,048 participants, 14,083 deaths occurred during 23 years of follow-up, with a significant association between dietary vitamin K1 and reduced CVD-related mortality when comparing the first quintile with the fifth quintile. The relationship was also found for subpopulations upon stratification into sex, smoking, diabetes, and hypertension. As vegetables are a major source of vitamin K1, it is possible that dietary vitamin K1 can be a marker of vegetable intake, which is known to protect against CVD. However, the associations persisted after adjustments for total vegetable intake [94].

There are also *intervention studies* addressing a possible role for a preventive effect of vitamin K in cardiovascular health. In an intervention study including 121 healthy participants, it was shown that a 3-year vitamin K1 (100 μg/day) supplementation period resulted in maintenance of vascular elasticity, in comparison with a 12% loss of elasticity in the placebo group [95]. In a placebo-controlled human trial of supplementation with vitamin K2 MK7 in postmenopausal women (*n* = 244), Knapen et al. showed that MK7 supplementation (180 μg/day) for 3 years significantly decreased pulse wave velocity, which is the gold standard for measuring arterial stiffness [96].

In 2019, Lees et al. [97] conducted a systematic review and meta-analysis of the connection of vitamin K with vascular health. They included 6 controlled clinical trials lasting for 6–36 months using PK 500–2000 μg/day or MK7 90–180 μg/day as supplementation. Three had vascular calcification as outcome (*n* = 407), and another three had vascular stiffness as outcome (*n* = 445); it was concluded that supplementation with vitamin K in comparison with placebo significantly reduced vascular calcification, while the reduction in vascular stiffness was not significant.

While most studies have used vitamin K2, Bellinge and co-workers in 2022 [98] investigated the effect of vitamin K1 and colchicine on vascular calcification in 154 patients with type 2 diabetes mellitus. After 3 months, neither vitamin K1 nor colchicine had any effect on coronary calcification, measured with 18F-NaF positron emission tomography; however, the intervention period was relatively short.

It appears from these reviewed studies that the approach of increasing vitamin K either by dietary means or through supplementation can delay or prevent CVDs, and that this effect apparently is obtained through optimized carboxylation of MGP, which chelates and redistributes calcium. However, large-scale, long-term supplemental trials are still needed to determine whether vitamin K_1_ or vitamin K_2_ reduces the risk of atherosclerosis including other CVDs and cardiovascular-related events like stroke, and the efficient dose range also remains to be settled.

## 8. Conclusions

Calcification in blood vessel walls is a pathological process that might accelerate the development of arterial stiffness and increase the risk of severe cardiovascular incidents. Vascular calcification commonly occurs in atherosclerosis, diabetes, chronic kidney disease, and aging. Although chelation therapy with EDTA may stabilize calcium precipitates in atherosclerotic plaques, calcium chelation with EDTA has negative impacts on bone by its mobilization of skeletal calcium. Today, intravenous EDTA infusions as therapy for CVD, which has been promoted by alternative medical centers, should be discouraged due to the risk of disruption of the physiological functions of several metal ions. It is also a puzzle that clinical trials on EDTA chelation therapy have never reported the important role of endogenous chelators. In this context, the therapeutic role of natural chelators such as VKDPs represents an option with low risk of side effects. Optimized intake of vitamin K has been shown to play a crucial role in preventing vascular calcification by regulating the carboxylation of specific VKDPs including MGP and osteocalcin, which are essential for physiological calcium metabolism, with beneficial effects on vascular health and bone mineralization.

MGP appears to be a potent vascular calcification inhibitor produced by vascular smooth muscle cells, chondrocytes, and other cells. In its carboxylated state, MGP binds calcium ions with high affinity and prevents their aberrant deposition, e.g., in the arterial wall. In contrast, dephosphorylated and undercarboxylated MGP (dp-ucMGP) has a low affinity for calcium ions and is inefficient in inhibiting vascular calcification. A high dp-ucMGP-to-total MGP ratio indicates a deficient vitamin K status and has been associated with an increased risk of vascular calcification. More research is needed regarding the optimal intake of vitamin K. Also, vitamin-K-dependent molecular mechanisms involved in vascular calcification, i.e., vitamin-K-dependent carboxylation and calcium chelation, and the role of SXR activation by MK4 and impact on inflammation need to be investigated. Potential therapeutic applications of vitamin K supplementation should be elucidated to develop effective interventions that can improve bone and vascular health.

Conclusively, further investigations are necessary to examine more in detail the roles of vitamin-K-dependent proteins in protection toward human atherosclerosis including mechanisms in vascular and valvular calcification, and to evaluate the effect of prolonged supplemental vitamin K as regard the risk of cardiovascular disease.

## Data Availability

The data used in this article are sourced from materials mentioned in the References section.
